# Decision-making in a population of spiking neurons shaped by dynamics of intrinsic noise

**DOI:** 10.1186/1471-2202-15-S1-P27

**Published:** 2014-07-21

**Authors:** Lydia Richardson, Jean-Philippe Thivierge

**Affiliations:** 1 Department of Biomedical Sciences, University of Ottawa, Ottawa, Ontario, Canada, K1N 9A8; 2 Department of Psychology, University of Ottawa, Ottawa, Ontario, Canada, K1N 9A8

## 

**Figure 1:**
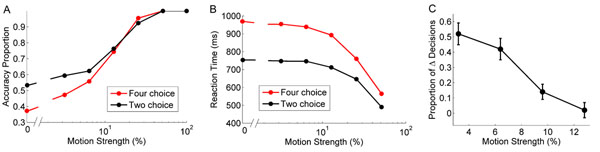
Speed & accuracy trade-off of a network using an internal noise source. **A.** Mean proportion of correctly selected choices relative to different motion strength values (0%, 3.2%, 6.4%, 12.8%, 25.6%, 51.2% & 100%) in a two (red) and four (black) choice discrimination task. Each dot represents the mean value of 1,000 trials. **B.** Mean reaction times (ms) of selected choices relative to the different motion strength values (0%, 3.2%, 6.4%, 12.8%, 25.6% & 51.2%). Each dot represents a mean value over 1,000 trials. **C.** The proportion of altered decisions relative to the motion strength (3.2%, 6.4%, 9.6% & 12.8%). The network is comprised of two pools of 50 neurons each integrating evidence in favor of a specific choice. On separate trials, a spike was added to a single neuron in favor of selecting the incorrect choice. The mean proportion of changed decisions was measured over 50 trials (1 spike/neuron for a total of 50 neurons) for each coherence value. Error bars are standard error.

Decision-making is a cognitive process involving a choice between multiple alternatives. In recent years, there has been a lot of progress about understanding the way decisions are carried out in the brain. Neurons accumulate both evidence and noise over time, by a summation of spikes, in favor of a specific choice. It remains unclear, however, what are the noise sources involved in brain processing during cognitive tasks. Theoretical models often use an external noisy term added to the network to create trial-to-trial variability by adding random fluctuating inputs to the membrane potential of neurons [1]. Here, we propose an alternative method of information processing by investigating the influence of intrinsic noise generated through the complex interactions between cells in a model of spiking neurons performing a decision-making task. A paradigm termed the random dot motion task [2], used in many perceptual tasks, was incorporated in the artificial neural network to measure the model’s performance with two- or four-choice alternatives. In this task, dots presented on a visual display move in a specific direction and an added percentage of dots are moving randomly. The network must discriminate among the net movement of dots. Figure 1A-B illustrates the speed/accuracy trade-off of the modeled network using an internal noise source when presented with a dot motion of different motion strength values (varying degree of difficulty according to the ratio of moving dots). The model was exquisitely sensitive to small changes in initial conditions, and the addition of a single post-synaptic spike was able to bias the network’s decision in a systematic and predictable fashion. Figure 1C shows that a decision can be altered when the network is presented with a single additional spike in favor of the opposite choice, especially at low motion strength values. In sum, results of the model capture experimental findings on the electrophysiology of decision-making and suggest a key role of intrinsic noise on cognitive processing.
